# The novel adamantane derivatives as potential mediators of inflammation and neural plasticity in diabetes mice with cognitive impairment

**DOI:** 10.1038/s41598-022-10187-y

**Published:** 2022-04-25

**Authors:** Iwona Piątkowska-Chmiel, Monika Gawrońska-Grzywacz, Łukasz Popiołek, Mariola Herbet, Jarosław Dudka

**Affiliations:** 1 Chair and Department of Toxicology, Faculty of Pharmacy, Medical University of Lublin, 8b Jaczewskiego Street, 20-090 Lublin, Poland; 2 Chair and Department of Organic Chemistry, Faculty of Pharmacy, Medical University of Lublin, 4A Chodźki Street, 20-093 Lublin, Poland

**Keywords:** Neuroscience, Endocrinology

## Abstract

Diabetes is a chronic disease leading to memory difficulties and deterioration of learning abilities. The previous studies showed that modulation of inflammatory pathways in the diabetic brain may reduce dysfunction or cell death in brain areas which are important for control of cognitive function. In the present study, we investigated the neuroprotective actions of newly synthesized adamantane derivatives on diabetes-induced cognitive impairment in mice. Our study relied on the fact that both vildagliptin and saxagliptin belong to DPP4 inhibitors and, contain adamantanyl group. Efficacy of tested compounds at reversing diabetes-induced different types of memory impairment was evaluated with the use of selected behavioural tests. The following neuroinflammatory indicators were also analyzed: neuroinflammatory indicators and the expression of genes involved in the inflammatory response of brain (*Cav1*, *Bdnf*). Our study demonstrated that new adamantane derivatives, similarly to DPP4 inhibitors, can restrict diabetes-induced cognitive deficits. We demonstrated that the overexpression of GLP-1-glucagon-like peptide as well as *Bdnf*, *Cav1* genes translate into central blockade of pro-inflammatory synthesis of cytokines and significantly improvement on memory performance in diabetes mice. Newly synthesized adamantane derivatives might have important roles in prevention and treatment of cognitive impairment by inflammatory events in patients with diabetes or related diseases.

## Introduction

Diabetes is one of the most prevalent chronic diseases which now is a serious health problem on a global scale. Statistical analyzes show that in 2030 the number of diabetic patients may reach 578 million and in 2045 as many as 700 million in the world^1^. Today we know that diabetes is a systemic disease that can increases the risk of cardiovascular problems, ophthalmic, and nephrological problems.

However, the one of the most dangerous complications of diabetes is the damage of the central nervous system. In development of cognitive impairment like as memory difficulties, deterioration of learning abilities or also psychomotor slowing may involve a multiple factors such as the hypoglycemic and hyperglycemic episodes, cerebrovascular alterations or insulin-signaling disorders^2^. It was proved that diabetes mellitus (DM) undoubtedly leads to cardiovascular impairment responsible for cognitive deficits^3^. Research showed that 20 to 40% of type 2 DM patients suffer from cerebrovascular diseases, including 45% of cases of dementia^3,4^. The studies performed in rat diabetes model (non-obese T2DN diabetic rats) proved that impaired autoregulation of the cerebral blood flow as well as blood brain barrier (BBB) leakage is essential for the development of diabetes-related and also age-related dementia^3^. In addition, in murine models it was also shown that age-related cerebrovascular dysfunction is associated with BBB disruption and it results in the process of neuroinflammation^5^. It must be highlighted that the microvascular endothelium is a significant source of paracrine mediators, including cytokines, chemokines and growth factors such as brain-derived neurotrophic factor (BDNF)^5^. The available studies indicate the essential role of inflammation in the development of neuronal injury in the brain^6^. Neuroinflammation mainly manifests as microglial activation and the increased release of inflammatory factors, such as tumor necrosis factor (TNF) and interleukins (IL-1*β*, IL-6), and free radicals, which can result in gradual dysfunction or death of cells in brain areas which are important in control of cognitive functions^6–8^. Recently, in the published article we confirmed a close relationship between hyperglycemia, hyperinsulinemia and neuroinflammation, and cognitive dysfunction in T2DM mouse model^9^.

In response to the previously mentioned pro-inflammatory factors, the body produces substances which prevent the development of inflammation in CNS. Research showed that glucagon-like peptide (GLP-1), which is both a peripherally expressed incretin and a centrally active neuropeptide, may promote neurogenesis, and it ameliorates cognitive deficits in preclinical models of neurodegeneration^10–13^. The neuroprotective effect of GLP-1 is most likely due to its action as a classic growth factor, which results in increased expression of genes related to cell growth, repair and replacement, increase of cellular metabolism, inhibition of apoptosis and reduction of inflammation^14^. Moreover, as it has been previously reported the increased availability of GLP-1 in the brain may have an inhibitory effect on the expression of genes encoding the pro-inflammatory cytokines: IL-6 and TNF-*α*^15^. Mice treated with a GLP-1 receptor agonists were observed to have an increase in the number of neurons in the hippocampus and, consequently, an improvement in their cognitive skills^16^. Additionally, GLP-1 mimetics have been shown to increase neurogenesis and to accelerate the formation of new neurons^13–17^, what suggest that greater availability of GLP-1 in central nervous system may be essential for its functionality.

Moreover, previous studies suggested that brain-derived neurotrophic factor level and activity of dipeptidyl peptidase 4 (DPP4) might both play an important role in the development of cognitive impairment^18^. Some of the studies confirmed the existence of a correlation between hyperglycemia and DPP4 activity, BDNF and cognitive impairment^19–23^. Besides this fact, research showed higher levels of DPP4 what may be exacerbated of inflammation and oxidative stress^24^.

Since several years, scientists become more interested in compounds with potential activity on central nervous system like the adamantane derivatives. It is dictated by the fact that an adamantane moiety is presented in the structure of many medicines and this system is considered to improve their pharmacological properties by increasing the lipophilicity and stability of the molecule^25^. Research proved that novel compounds incorporating an adamantane moiety have neurogenic and neuroplastic properties and enhance cognition in normal adult mice. Therefore, they may be the attractive candidates for the development of pro-cognitive drugs used in the prevention and treatment of learning and memory disorders and neurodegenerative diseases^26^. In addition to this, some of adamantane derivatives, like amantadine or memantine, are currently used to treat neurological disorders in diseases such as Parkinson^27^ and Alzheimer’s^28^ or after traumatic brain injury^29^ or to severe cerebral hemorrhage^30^. The adamantane moiety is also present in the structure of known anti-diabetic drugs from the group of dipeptidyl peptidase 4 inhibitors (DPP4Is) such as vildagliptin or saxagliptin.

Latest research reports suggest that the beneficial effects of drugs which contain adamantanyl group may be related with the ability of these molecules to control neuroinflammation pathways^10,21,31,32^. The performed studies also showed that modulation of inflammatory response of brain by these compounds may contribute to alleviation of neuroinflammation and limitations of neurological deficits^33–36^. Sulkowski et al*.*^37^ confirmed the anti-inflammatory effect of both amantadine and memantine in experimental model of allergic encephalomyelitis (EAE) and multiple sclerosis model. These drugs limited neurological deficits in rats by decreasing expression of interleukins like IL-6, IL-1*β*, and TNF-*α*.

Several studies also indicated that DPP4 inhibitors may prevent the progression of diabetes and improve the cognitive impairment associated with the disease by a modulation of inflammatory pathways^38–42^. El-Sahar et al*.*^43^ noted that sitagliptin reduces inflammation in the brains of diabetic animals by reducing the levels of TNF-*α* and IL-6. The research performed by Újhelyi et al*.*^44^ confirmed the anti-inflammatory effects of two DPP4 inhibitor derivatives: sitagliptin and vildagliptin in mouse model of inflammation. In the above-mentioned studies, the tested drugs effectively reduced the level of pro-inflammatory markers in the hippocampus and in inflamed tissues. Satoh-Asahara et al*.*^45^ also proved that therapy with DPP4 inhibitors not only lowered the level of pro-inflammatory markers such as TNF-*α*, but it also increased the levels of anti-inflammatory cytokines what ultimately led to the reduction of inflammation in the blood serum of patients with diabetes. In turn, Makdissi et al*.*^34^ showed that sitagliptin, apart from a significant reduction of TNF-*α* and IL-6 level, simultaneously increased the fasting blood level of GLP-1 in obese diabetic patients. Thus, inhibition of neuroinflammation may provide cognitive protection. In clinical trials with patients suffered from type 2 diabetes and mild cognitive impairment (MCI) and treated for 2 years with DPP4 inhibitors, i.e. vildagliptin, sitagliptin or saxagliptin, a significant improvement in cognitive activity was noted in comparison with patients treated with sulfonylureas^35^.

Furthermore, the analysis of available studies showed that levels of gene expression of *Bdnf* and *Cav1* in brain may have also a significant effect on the modulating of neuroplasticity^46–50^. The studies have shown that chronic hyperglycemia decreases *Cav1* expression in the neurons of diabetic rats, what results in the development of neuroinflammation and the increase of tau phosphorylation, and consequently accelerates neurodegeneration^48–50^. Bonds et al*.*^50^ found that the low expression of *Cav1* in brains of diabetic mice promotes their impairment learning and memory process. Additionally, *Cav1* overexpression in these mice is correlated with their cognitive activity. Research performed by Wu et al*.*^48^ showed that overexpression of *Cav1* decreases HGC-induced tau hyperphosphorylation in the hippocampal primary neurons. Qin et al*.*^18^ confirmed the relationship between the decrease of peripheral blood BDNF level and the progression of Alzheimer’s disease (AD) or MCI. Beside this, Han et al*.*^46^ showed that *Bdnf* overexpression inhibits hyperglycemia-induced microglial activation and it reduces neuroinflammation in the hippocampus of type 1 diabetic mice. Thus, the modulation of the expression of genes involved in the inflammatory response of brain (*Cav1, Bdnf*) may contribute to alleviation of neuroinflammation and the progression of cognitive disorders.

These encouraging findings have prompted us to undertake research on new adamantane derivatives. In this manuscript we reported the synthesis and biological evaluation of novel adamantane derivatives in CNS of mice with type 2 diabetes. In our work we examined whether the tested compounds are able to limit the neuroinflammation and reverse cognitive dysfunctions in diabetic mice. The effectiveness of the tested compounds in reversing different types of diabetes-induced memory impairment was assessed using the Y-maze, a new object recognition test, and a passive avoidance test.

In this study we postulated that anti-inflammatory actions of tested compounds may arise from DPP4 inhibition. To probe for a putative link between modulation of protein levels of DPP4 and GLP-1 and inflammation, we assessed in the prefrontal cortex of mice the following parameters: IL-1*β*, IL-6, tumor necrosis factor-α (TNF-*α*), and glucagon-like peptide (GLP-1) as well as the expression of genes involved in the inflammatory response of brain (*Cav1, Bdnf*).

## Materials and methods

### Chemistry

All reagents and solvents used in this research were purchased from Sigma-Aldrich (Munich, Germany) and Merck Co. (Darmstadt, Germany) and used without further purification. Thin layer chromatography (TLC) on plates covered with silica gel (aluminum oxide 60 F-254, Merck Co.) was used to check the purity of the obtained compounds and to monitor the progress of the reaction. Chloroform-ethanol mixture in the 10:1 (v/v) ratio was used as the mobile phase. The spots were detected by irradiation with UV light at a wavelength of λ = 254 nm. ^1^H NMR and ^13^C NMR spectra were recorded on the Bruker Avance 300 and 600 apparatus (Bruker BioSpin GmbH, Germany). The melting points of the obtained compounds were measured with a Fisher-Johns apparatus (Fisher Scientific, Germany), and presented without any correction. The elemental analysis was determined by the Perkin Elmer 2400 series II CHNS/O analyzer (Waltham, MA, USA), and the results were within ± 0.4% of the theoretical value.

#### Procedure of synthesis of adamantane derivatives (2, 3)

The synthesis was conducted on the basis of procedure reported earlier^51^ and applied for the synthesis of hydrazide-hydrazones^51–54^. 0.01 mol of 3-amino-1-adamantanol (**1**) was placed in the round bottom flask and ethanol (5 mL, 96%) was added. The mixture was heated under reflux until 3-amino-1-adamantanol (**1**) was dissolved. Then 0.011 mol of appropriate aromatic aldehyde was added and the solution was heated under reflux for 3 h. After that the mixture was allowed to cool and the formed precipitate was filtered off, dried and re-crystallized from ethanol (96%).

### Drugs and chemicals

In the experiment we used: crystalline fructose (Biomus, Lublin, Poland), streptozotocin (≥ 98% HPLC, Sigma-Aldrich, Munich, Germany), vildagliptin (50 mg, Galvus, Novartis Pharma GmbH, Nurnberg, Germany), saxagliptin (5 mg, Onglyza, AstraZeneca Pharma Poland Sp. z o.o., Poland), and sodium citrate and citric acid (Biomus, Lublin, Poland) which were used to prepare the citrate buffer 0.01 M, pH = 4. We have also used novel adamantane derivatives numbered as compound **2** and **3** which were synthesized by dr hab. Łukasz Popiołek (Chair and Department of Organic Chemistry, Medical University of Lublin, Lublin, Poland). Sterile aqueous solutions of tested compounds were prepared *ex tempore* (Aqua pro injectione, Baxter, Warsaw, Poland).

### Animals

In the experiment, we used 48 adult, seven-eight-week-old CD-1 males mice (output weight of 20–23 g). Mice were obtained from a licensed breeder’s animal facility, Experimental Medicine Centre (EMC), Medical University of Lublin, Poland, (077—EMC number in Lublin in the Breeders' Register kept by the Minister of Science and Higher Education, (Poland)). They were bred in controlled conditions, in accordance with the legal regulations of the Regulation of the Minister of Agriculture and Rural Development of December 14, 2016 (Journal of Laws, 2139). Mice were bred and divided into groups of 2–3 per cage. A standard diet, water, or fructose solution was available *ad libidum*. Body weight was measured weekly. Behavioural tests were conducted in a separate room. Procedures involving rodents and their care in all the experiments of our study were approved by the Local Ethics Committee at the University of Life Science in Lublin (No. 43/2018, Lublin, Poland). Procedures were performed in accordance with binding European standards related to the experimental studies on animal models (Act from January 15, 2015 on the Protection of Animals Used for Scientific or Educational Purposes; Directive 2010/63/eu of the European Parliament and of the council of 22 September 2010 on the protection of animals used for scientific purposes). All activities were carried out by qualified staff, the animals were under the constant supervision of the veterinarian; all efforts were made to minimize suffering.

### Experimental procedures

#### Induction of type 2 diabetes in mice

The experiment was divided into several stages. In the first step, adult male mice were randomly divided into 6 groups: (1) control group (non-diabetic mice—CTL); (2) test group (diabetes-induced mice—DM); (3) vildagliptin-treated DM mice; (4) saxagliptin-treated DM mice; (5) DM mice treated with compound **2**; 6) DM mice treated with compound **3**. Thereafter, all groups, except the control group, were administered 20% aqueous solution of fructose *ad libidum* for 4 weeks and then for the next 5 days they were administered intraperitoneal injection of freshly prepared solution of streptozotocin (STZ), (1 × daily 40 mg/kg) in 0.01 M cold citrate buffer pH 4.5. During this time, the control group was administered citrate buffer alone in the same concentration.

The induction of type 2 diabetes in mice was carried out as described earlier^9^. For a verification of the type 2 diabetes mellitus (T2DM) model, the blood levels of glucose and insulin were determined and HOMA-IR index was calculated.

In the next stage, all experimental groups with confirmed diabetes (except group 2) received orally 1 × daily for the next 14 days aqueous solutions of tested compounds (0.01 ml/kg body weight): adamantane derivatives: compound **2** and **3** (50 mg/kg; DM-compound **2**; DM-compound **3**), vildagliptin (20 mg/kg; DM-Vil) or saxagliptin (10 mg/kg; DM-Sax). At the same time, mice from control and diabetes-induced groups received a suitable vehicle i.e. physiological saline.

Bearing in mind the fact that the level of spontaneous locomotor activity of animals may have an impact on the results of memory tests, after 2 weeks of treatment with the tested compounds, we carried out the open-field test (OFT). Next, the mice were subjected to behavioural tests evaluating their cognitive function in the following order: the Y-Maze Spontaneous Alternation Test, the Y Maze Novelty Preference Test (NPT), Novelty Object Recognition Test (NOR test), and Passive Avoidance Test (PA test). In the final stage of the experiment, the mice were sacrificed by decapitation, and their brains were isolated. The GLP-1, IL-1*β*, IL-6 and TNF*α* levels were determined in the prefrontal cortex of mice. While, the expression levels of *Cav1* and *Bdnf* genes were analysed in the hippocampus of mice.

#### Behavioural tests

All behavioural tests were carried out under controlled environmental conditions, such as temperature, humidity, and light intensity (dim illumination). In order to avoid possible circadian modifications of the test results, all experiments were carried out between 9.00 am and 11.00 am. In order to eliminate olfactory cues, all apparatuses were systematically cleaned. Behavioural tests were performed by two blind observers in real time.

##### The open-field test (OFT)

Open-Field Test was carried out to measure the levels of spontaneous locomotor activity of animals. The test was carried out in the box constructed of natural wood with the floor dimensions of 40 × 40 cm and a wall height of 35 cm. Mice were individually placed at the centre of the arena and allowed to explore the arena, freely and uninterrupted, for 5 min. The assessment of spontaneous locomotor activity was based on the time measurement the animals did move (s). Test was performed according to the procedure described by Hall^55^.

##### The Y-maze spontaneous alternation test

The Y-maze was used to conduct the spontaneous alternation task and novelty preference test. The Y-maze was made of grey non-reflective and odour resistant material which was easy to clean. The walls of the maze were opaque. Maze was also equipped with three partitions to easily separate each arm.

Y-maze spontaneous alternation behaviour is a test used to measure spatial working memory during exploratory activity of animals. The test was carried out in a three-arm labyrinth, which arms had equal size and were labelled as A, B, and C, respectively. Test was performed according to the procedure described by Hughes^56^. Each arm was 34 cm long, 8 cm wide, and 14.5 cm high and was oriented at an angle of 120° from the other two.

Each mouse was placed at the end of one arm and allowed to move freely through the maze for 5 min. The number of arm entries and the number of the sequence (e.g. ABABCC) were recorded manually for each mouse. An arm entry by mouse was judged to be completed when the hind paws of them were completely placed in an arm. The parameters measured included number of mouse entries to three different arms consecutively-spontaneous alternation performance (SAP) (i.e. ABC, ACB, BCA, BAC) and number of mouse visit other arms and return to the same arms—alternate arm returns (AAR) (i.e. ABA, ACA, BAB) and visit of mouse in the same arm repeatedly—same arm return (SAR) (i.e. AA, BB, CC).

Percent of spontaneous alternation performance was calculated as [(number of alternations)/(total arm entries − 2)] × 100.

The maze was cleaned with 70% solution of ethanol (v/v) after each mouse, so as to avoid olfactory cues.

##### The Y maze novelty preference test (NPT)

**Scheme 1:**
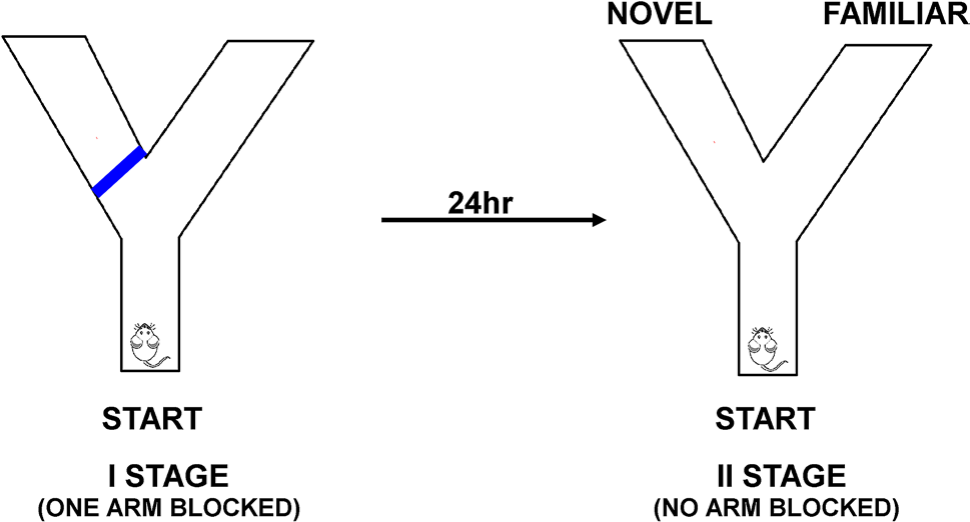
The Y-Maze Novelty Preference Test (NPT) protocol.

The Y-Maze novelty preference test was used for evaluation of spatial novelty-objected cognition memory. The procedure of test was carried out based on modifications of the Melnikova et al*.* method^57^. This test was conducted in the same Y-maze as spontaneous alternation test. This test consisted of two stages. During the first stage of test, one arm of the Y-maze was closed by the a guillotine door (in blue) (Scheme 1). Next, each mouse was individually placed in the maze in the start arm, and it was allowed to explore two arms for 5 min (the Start and Other arm) of the Y-maze, while entry into the third arm was blocked. Next, after the end of this stage, the mouse was removed from the maze and placed back into their home-cage. After 24-h delay, the second phase of the experiment began. The novel arm of Y-maze was opened. During the 5 min of observation, each mouse had free access to all three arms of Y-maze.The number of entries of a mouse to the arms of Y-maze was recorded. Mice without spatial memory deficits should prefer the previously unvisited, novel arm over the two familiar arms. Preference novelty (PN) index was calculated as a ratio of the number of entries of a mouse to the novel arm to the number of entries to other arms of maze. Score greater than 0.5 indicate a novelty preference. Each of the arms of the Y maze was cleaned with 70% ethanol solution and wiped dry with a paper towel before next mouse.

##### The novel object recognition test (NOR)

Apparatus: The test was carried out in a square chamber (40 cm × 40 cm × 40 cm) made of white-coloured wood. Wooden cubes in the shape of a oval, rectangular or triangular pyramid (4 cm × 4 cm × 6 cm) were used as objects. The procedure: the NOR test evaluates natural preference of mice to recognize a novel object in the environment. It is useful to study short-term, intermediate-term, and long-term memory, through manipulation of the retention interval, i.e., the amount of time elapsed between the familiarization phase and the test phase. The test consists of three stages: habituation, training, and testing. The procedure of a test was performed as described in the previous studies with modifications^9^.

**Scheme 2:**
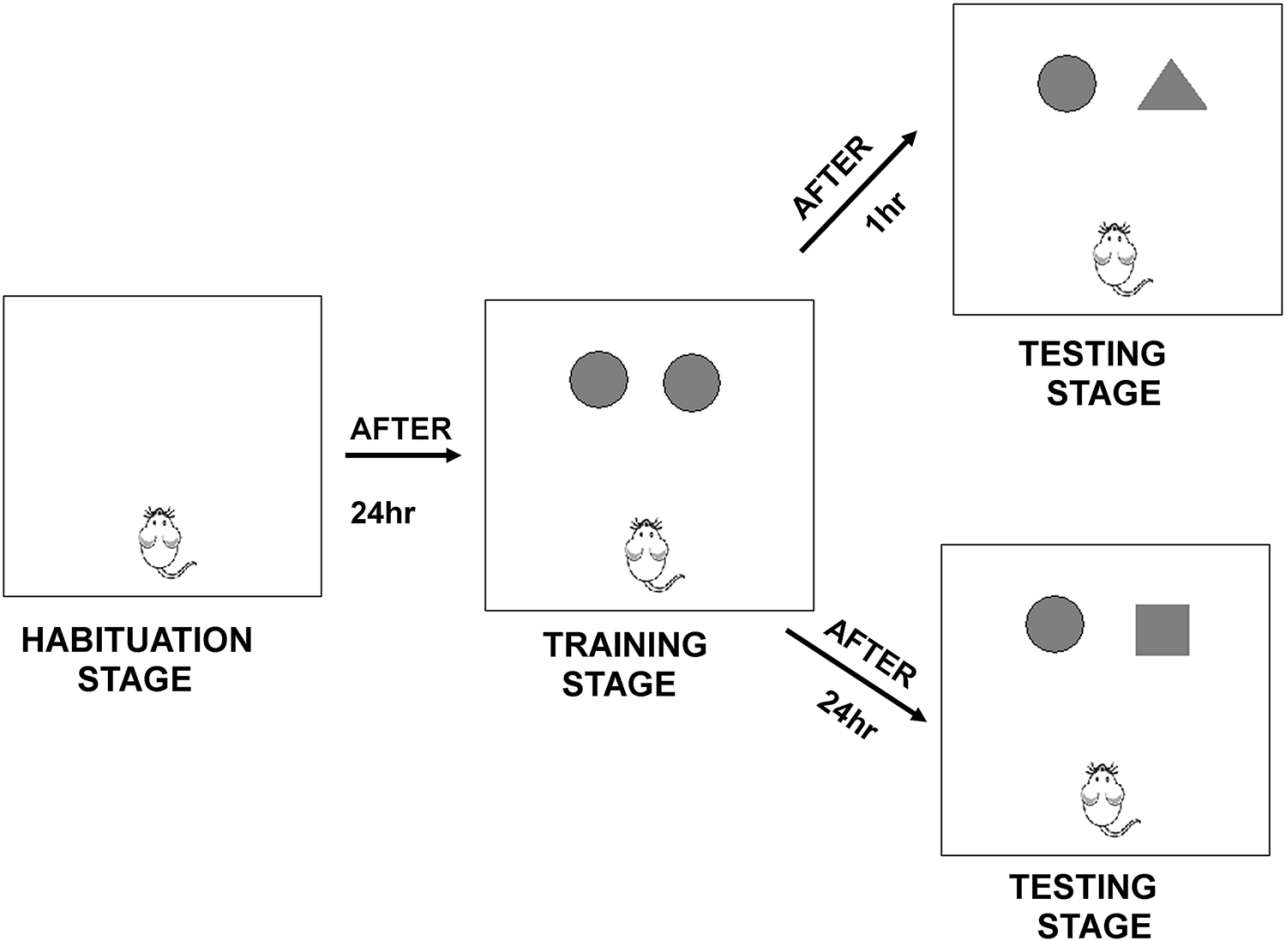
The scheme illustrates phases of the novel object recognition (NOR) test.

During habituation sessions, the mice were individually habituated to the anempty box for 10 min (on which the test will be carried out) and then they were removed from the arena and placed in a home-cage (Scheme 2). After twenty-four hours the animals were placed again in the arena for 5 min with two identical objects (oval white blocks)—training stage. We observed the number of mouse interactions with individual blocks for 5 min. After 1 h break the testing phase begins. The experimental context is not drastically different during the training and the testing phases.

In the testing stage of the experiment, one of the previously learned objects was changed to a new, unknown object—triangular block about the same colour (white) and size (weight, height and width). Sets of objects were chosen based on preliminary experiments that indicated that they were similarly preferred by mice. During the 5 min of observation, the time of interactions of mice with individual blocks was recorded (directing the head towards the object, with its neck extending and vibrissae moving). Both turning around and sitting on the objects were not considered exploratory behaviors.

24 h later one of the familiar objects was replaced and a novel object introduced, and the test repeated as previously. During 5 min of observation, the number of exploring behaviors of each object by a mouse was recorded. According to the innate curiosity of animals, healthy mice should spend more time on exploration of a new object.

The preference index (%PI) was calculated as exploration time of the novel object (Nov) divided by the total exploration time of both, novel (Nov) and familiar (Fam) objects [%PI = TN/(TN + TF) × 100]. Similarly, this calculation can be applied when both objects are identical, in the training session, but here the mathematic formula will be exploration time of the right object (R) divided by the total exploration time of both, right (R) and left (L) objects [%PI = R/(R + L) × 100]^58^.

The cage and blocks were thoroughly cleaned between each use with 70% ethanol.

##### Passive avoidance test (PA)

The passive avoidance test was employed for the measurement of learning and memory^9^. The PA test allows the examination of both, short-term memory, (developing within a few seconds or minutes after single training session) and long-term memory (lasting for at least 24 h). The test was based on the natural tendency of rodents to prefer the dark place.

**Scheme 3:**
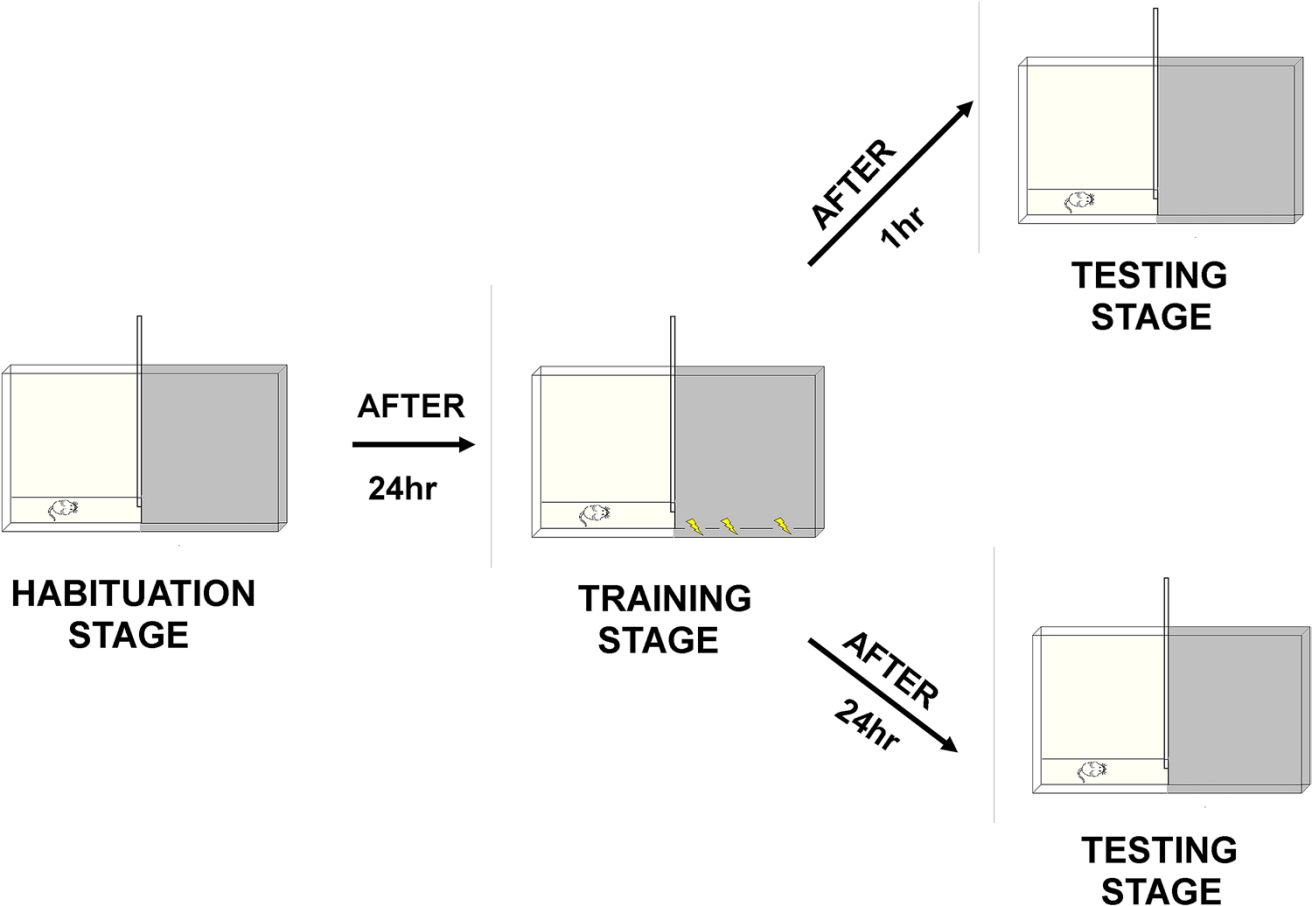
A scheme illustrates phases of the passive avoidance test.

*Apparatus* For the test, we used the special cage, divided into equal parts by a partition with guillotine doors. One part of a cage was illuminated, the other—darkened. The dark compartment has a removable cover made of the same material what a cage. Procedure: in order to habituate the mice to the apparatus, they were exposed to habituation test. The animals were placed individually into a lighted section of the apparatus and next the guillotine door was opened. Considering the natural tendency of the rodents to the dark environment, mice readily passed to enter the dark compartment. After the animals entered the dark compartment, the door was closed and then after 30 s they were taken from the dark compartment and placed in their house-cage. After that, on the following days training and testing sessions were carried out (Scheme 3). On the 1st day, one hour before the training session, mice were administered with compounds orally, while control animals received physiological saline. Then, each mouse was individually placed in an illuminated zone. According to the natural predisposition, the mouse preferred the darkened area. When the animal crossed into the dark chamber with all four paws, it was administered a mild foot shock (0.6 mA, 2 s duration). The latency time for entering the dark compartment was recorded for 180 s. If the mouse failed to enter the dark compartment at the time indicated above, it was placed into this the dark part, the door was closed, and the electric foot shock was delivered to the animal.

Testing sessions took place one hour and twenty-four hours after the training session (the electrical stimulus was no longer used). Each mouse was placed again in the illuminated compartment and after a 30 s adaptation, the door between the compartments was raised and the time taken to re-enter animal the dark compartment was recorded. The latency to enter the dark chamber was recorded up to 180 s. If the mouse did not enter the dark compartment within 180 s, the session was terminated and a latency of 180 s was assigned. The longer the delay was in the animals’ transition to the dark part of the apparatus, better their memory was. A cage was thoroughly cleaned with 70% solution of ethanol (v/v) before and between each use to reduce odour stimuli.

#### Biochemical determinations

##### Tissue material collection and preparation of homogenates of prefrontal cortex from mice

The samples of biological material from all groups of animals were collected with the use of standardized protocol. 24 h after the behavioural tests, the mice were sacrificed by decapitation while keeping animals for the last 4–6 h of fasting. After decapitation, brains were carefully removed. Then, the prefrontal cortex and hippocampus were isolated on ice from brain of every animal. After isolating, the tissue samples were rinsed in ice-cold PBS thoroughly and weighed, next they were cut into smaller pieces and homogenized on ice in PBS (w:v = 1:2). The tissue homogenates were cleared by centrifugation at 10,000×*g* for 5 min at 4 °C. The concentration of tested parameters in brain samples were normalized to the total protein concentration which was determined by the Bradford method^59^.

##### Quantitative determination of GLP-1 and DPP4 protein level in prefrontal cortex of mice

The levels of Glucagon-Like Peptide 1 (GLP-1) and Dipeptidyl-Peptidase 4 (DPP4) protein were determined by ELISA method using mouse kits (GLP-1, Cloud-Clone Corp., USA; DPP4/CD26, Cloud-CloneCorp., Wuhan, China) according to the manufacturer's instructions (S1).

##### Quantitative determination of cytokine IL-6, IL-1β and TNF-α protein level in prefrontal cortex of mice

The levels of cytokines in the prefrontal cortex were determined using ready-made for mice ELISA Kits (Interleukin IL-1*β*, IL-6 and TNF-*α*, Cloud-Clone Corp., Houston, TX, USA). We performed the determinations in accordance with the manufacturer’s instructions (S2).

##### Analysis of mRNA Bdnf and Cav1 expression in mice hippocampus—RT-qPCR

The mRNA isolation, selection of reference genes and expression levels of *Bdnf* and *Cav1* genes involved in the inflammatory response in hippocampal tissues of mice were performed in the same way as in the article recently published by Piatkowska-Chmiel et al*.*^9^. The primer sequences for the genes analyzed are presented in Table 1.

**Table 1:** The table shows the data on used primers: gene symbols, assay IDs, gene names, GenBank references sequence accession numbers andy amplicon lengths (bp).

Gene symbol	Assay ID	Gene name	RefSeq	Amplicon length (bp)
*Bdnf*	AB ID: Mm01334047_m1	Brain derived neurotrophic factor	NM_007540.4	105
*Cav1*	AB ID: Mm00483057_m1	Caveolin 1	NM_001243064.1, NM_007616.4	67
*Pgk1*	AB ID: Mm01225301_m1	Phosphoglycerate kinase 1	NM_008828.3	60
*Tbp*	AB ID: Mm00446974_m1	TATA box binding protein	NM_013684.3	105

*AB ID* applied biosystems TaqMan GENE EXPRESSION ASSAY ID.

### Statistical analysis

The differences between the studied groups were analysed using one-way ANOVA or repeated-measures ANOVA, followed by the Tukey's test for multiple comparisons, using GraphPad Prism (version 8.0). Pearson’s coefficient was used for an assessment of correlation. The minimal level of significance was identified at *p* < 0.05 (95% confidence interval limits). The results are presented as mean values ± standard deviation (SD).

### Institutional review board statement

The study was conducted according to the guidelines of the ARRIVE, and was approved by the Ethics Committee for Animal Research (No. 43/2018, 26 March 2018).

## Results

### Chemistry

**Scheme 4:**
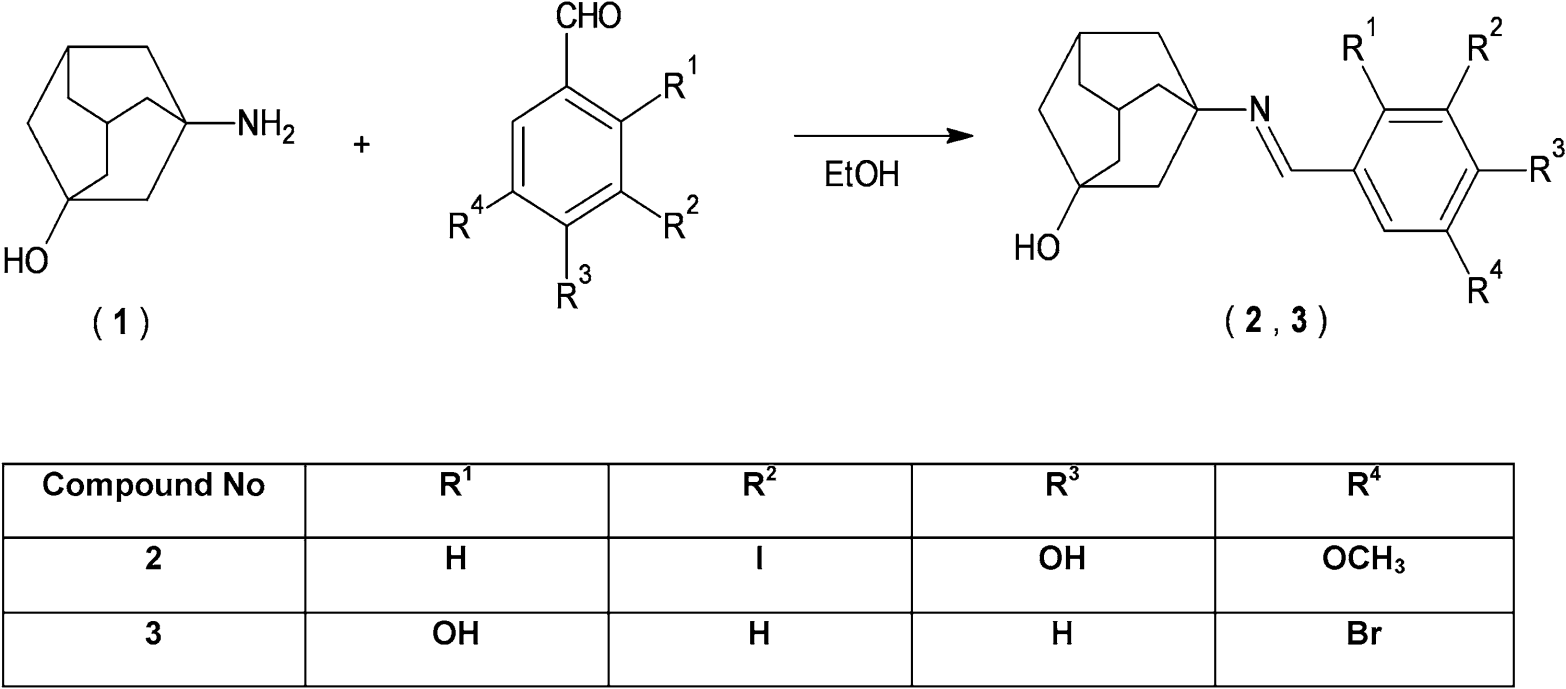
Synthesis of adamantane derivatives.

New derivatives of adamantane were synthesized on the basis of condensation reaction of 3-amino-1-adamantanol (**1**) with two different aromatic aldehydes (Scheme 4). Yield of the reaction was 68% for derivative **2** and 72% for **3**. Chemical structure of obtained adamantane derivatives was established on the basis of the analysis of ^1^H NMR and ^13^C NMR spectra.

Analysis of obtained ^1^H NMR and ^13^C NMR spectra enabled to clearly confirm the chemical structure of obtained novel adamantane derivatives.

In the ^1^H NMR spectra of compounds **2** and **3** characteristic singlet signals at δ 7.99 ppm (compound **2**) and 8.53 ppm (compound **3**) were found. The presence of the signal for = CH group confirmed successful synthesis of adamantane derivatives by the condensation reaction.

Whereas, in the ^13^C NMR spectra signals for carbon atom of = CH group appeared at δ 154.02 ppm (compound **2**) and 160.61 ppm (compound **3**). Other signals in ^1^H NMR and ^13^C NMR spectra for aliphatic and aromatic fragments of obtained molecules were found at expected values of chemical shift.

### Physico-chemical data of adamantane derivatives (2, 3)

#### 3-{[(4-hydroxy-3-iodo-5-methoxyphenyl)methylidene]amino}tricyclo[3.3.1.1^3,7^]decan-1-ol (2)

Yield: 68%; M.p.: 196–198 °C; ^1^H NMR (300 MHz, DMSO*-d*_*6*_): 1.50–1.68 (m, 12H, 6xCH_2-adamantane_), 2.22–2.24 (m, 2H, 2xCH_adamantane_), 3.74 (s, 3H, OCH_3_), 4.66 (s, 1H, OH), 7.30 (s, 1H, ArH), 7.77 (s, 1H, ArH), 7.99 (s, 1H, = CH), 9.29 (s, 1H, OH); ^13^C NMR (150 MHz, DMSO*-d*_*6*_): 30.15 (CH_adamantane_), 30.64 (CH_adamantane_), 43.89 (3xCH_2-adamantane_), 48.74 (3xCH_2-adamantane_), 53.29 (OCH_3_), 56.49 (C_adamantane_), 68.11 (C_adamantane_), 97.12, 114.18, 129.69, 133.93, 148.27, 150.73 (6C_ar_), 154.02 (= CH).

#### 3-{[(5-bromo-2-hydroxyphenyl)methylidene]amino}tricyclo[3.3.1.1^3,7^]decan-1-ol (3)

Yield: 72%; M.p.: 112–114 °C; ^1^H NMR (300 MHz, DMSO*-d*_*6*_): 1.51–1.66 (m, 12H, 6xCH_2-adamantane_), 2.24–2.25 (m, 2H, 2xCH_adamantane_), 4.67 (s, 1H, OH), 6.78–6.81 (d, 1H, ArH, *J* = 9 Hz), 7.40–7.44 (m, 1H, ArH), 7.69–7.70 (d, 1H, ArH, *J* = 3 Hz), 8.53 (s, 1H, = CH), 14.47 (s, 1H, OH); ^13^C NMR (150 MHz, DMSO*-d*_*6*_): 30.58 (CH_adamantane_), 34.94 (CH_adamantane_), 41.84 (3xCH_2-admantane_), 44.39 (2xCH_2-adamantane_), 50.50 (CH_2-adamantane_), 60.33 (C_adamantane_), 68.06 (C_adamantane_), 108.84, 119.87, 120.60, 134.34, 135.13 (5C_ar_), 160.61 (= CH), 161.71 (C_ar_).

### The effect of tested compounds on the spontaneous locomotor activity in diabetic mice in open field test

**Figure 1:**
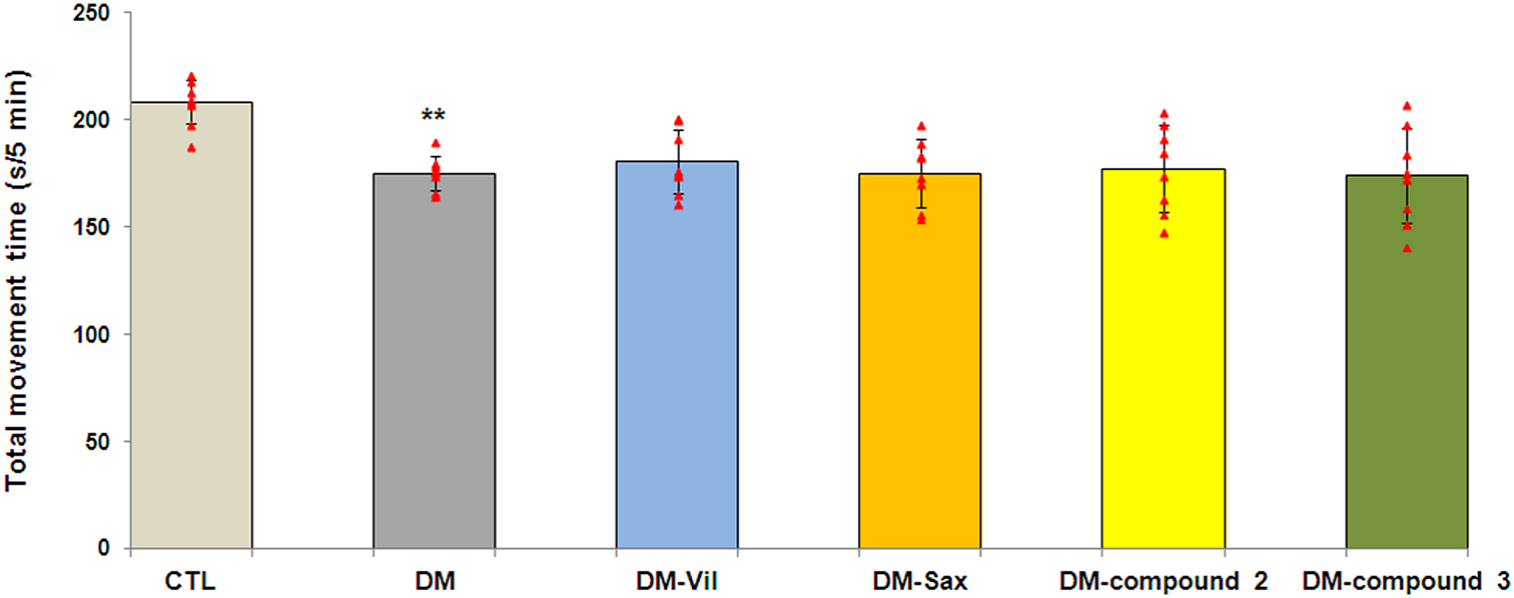
Spontaneous locomotor activity of mice in the open field test. Total movement time of mice (in seconds), over the whole area of the box, during 5 min of observation. Bars in figure illustrates experimental groups—*CTL* non-diabetic mice, *DM* diabetic mice, *DM-Vil* vildagliptin-treated mice [20 mg/kg, *po*], *DM-Sax* saxagliptin-treated mice [10 mg/kg, *po*], *DM-compound 2* compound 2-treated mice [50 mg/kg, *po*], *DM-compound 3* compound 3-treated mice [50 mg/kg, *po*], respectively. Each column represent means ± SD (n = 8). One-way ANOVA, followed by Tukey's post hoc test was used. **p < 0.01 as compared with healthy control group.

In the open field test, 14-days treatment with new adamantane derivatives or DPP4 inhibitors did not affect the locomotor activity of mice when compared to diabetes mice. As shown on Fig. 1, the average movement time of mice was similar in each treated group (*p* > 0.05).

### The effect of tested compounds on diabetes-induced memory impairments

**Figure 2:**
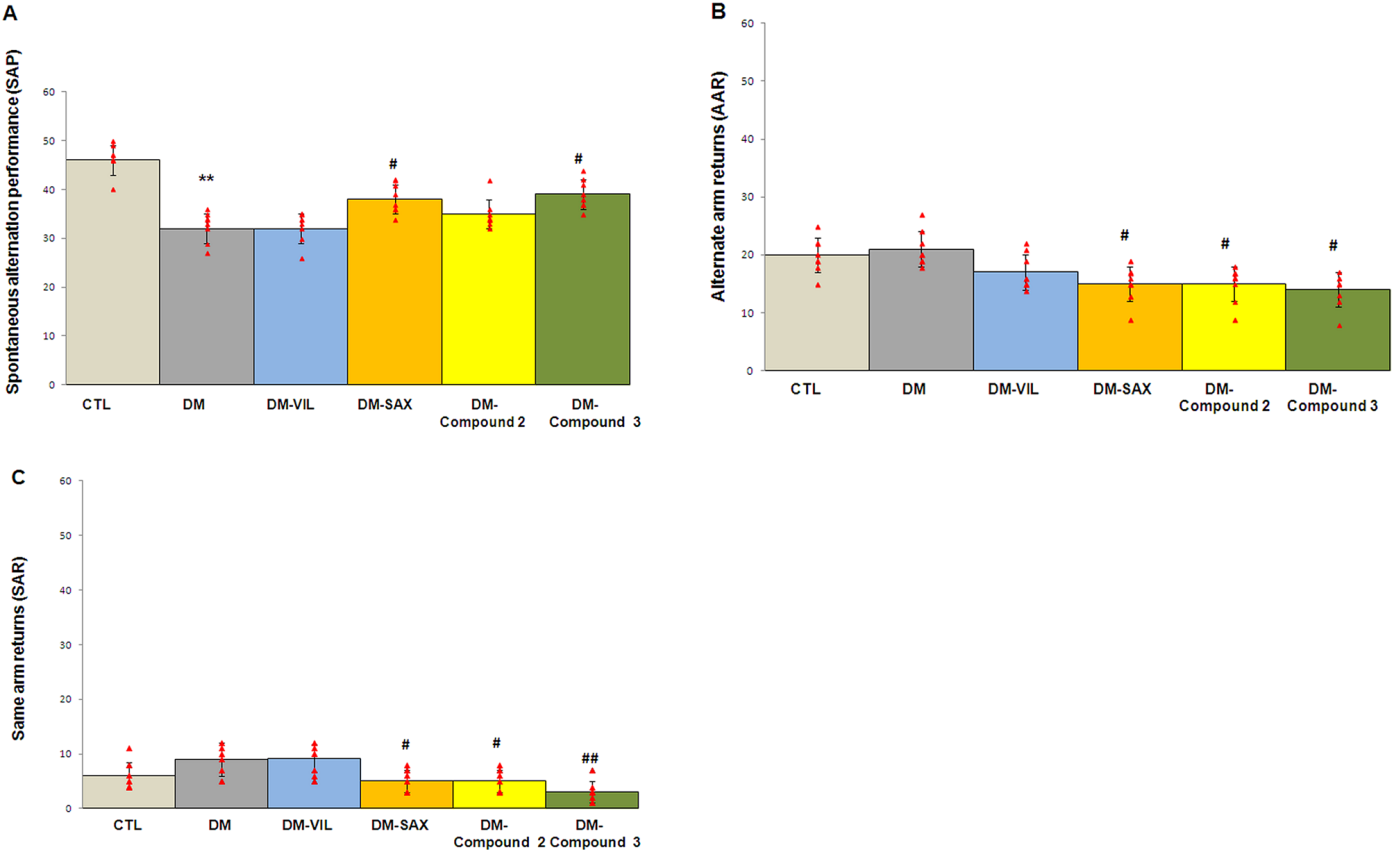
Novel adamantane derivatives (**2**, **3**) and DPP4 inhibitors ameliorate the working spatial memory impairment of diabetic mice in Y-maze test. (**A**) The spontaneous alteration performance (SAP) was defined as visit of animal in three different arms consecutively (i.e. ABC, ACB, BCA, BAC); (**B**) alternate arm returns (AAR) was defined as visit of mouse in the other arms and return to the same arms (i.e. ABA, ACA, BAB); (**C**) same arm returns (SAR) was defined as visit of mouse the same arm repeatedly (i.e. AA, BB, CC); The frequency of the behaviors: spontaneous alternation performance (SAP), alternate arm returns (AAR) and same arm returns (SAR) in all groups was counted and is presented as means ± SD (n = 8). One-way ANOVA, followed by Tukey's post hoc test was used. Bars in figure illustrate experimental groups—*CTL* non-diabetic mice, *DM* diabetic mice, *DM-Vil* vildagliptin-treated mice [20 mg/kg, *po*], *DM-Sax* saxagliptin-treated mice [10 mg/kg, *po*), *DM-compound 2* compound 2-treated mice [50 mg/kg, *po*], *DM-compound 3* compound 3-treated mice [50 mg/kg, *po*], respectively. **p < 0.01 as compared with healthy control group. ^#^p < 0.05; ^##^p < 0.01 compared with diabetic mice group.

The working, spatial memory performance was assessed by recording spontaneous alteration behaviour in Y-maze test. Both, diabetes and tested compounds affected spatial memory performance in the Y-maze (Fig. 2A–C). In the group of diabetes mice, a reduction of the spontaneous alternation in the Y-maze task was observed when compared to control healthy group (95% CI [29.933, 35.067], **p < 0.01; F = 24.117; Fig. 2A). 14-days treatment of mice with derivative **3** (50 mg/kg, *po*) or saxagliptin (10 mg/kg, *po*) effectively increased percentage of spontaneous alteration and improved memory impairment caused by diabetes.

Group of animals treated for 14 days with compound **3** or saxagliptin showed significantly enhanced spontaneous alternation performance (SAP) compared to the diabetic group (95% CI [36.619, 41.631], ^#^*p* < 0.05, F = 24.117; 95% CI [35.810, 40.940], ^#^*p* < 0.05, F = 24.117; Fig. 2A, respectively). The groups of animals treated with vildagliptin or derivative **2** exhibited no significant improvement with respect to the number of spontaneous alternation performances (SAP) (*p* > 0.05).

The differences in the numbers of alternate arm returns (AAR) and the same arm return (SAR) were not statistically significant in diabetic mice when compared to control mice (p > 0.05; Fig. 2B,C). However, in the groups of mice treated with derivatives **2** and **3** or saxagliptin, the significant decreases in number of AARs were noted when compared to the diabetic group (95% CI [12.472, 17.528], ^#^*p* < 0.05, F = 7.500; 95% CI [11.552, 16.448], ^#^*p* < 0.05, F = 7.500; 95% CI [12.579, 17.671], ^#^*p* < 0.05, F = 7.500; Fig. 2B, respectively). The saxagliptin and tested derivatives (**2**, **3**) also significantly reduced the number of returns by mice to the same maze arm repeatedly (95% CI [3.389, 6.611], ^#^*p* < 0.05, F = 8.283; 95% CI [3.389, 6.611], ^#^*p* < 0.05, F = 8.283; 95% CI [1.389, 4.611], ^##^*p* < 0.01, F = 8.283; Fig. 2C, appropriately).

14 days of vildagliptin treatment did not significantly AAR or SAR (*p* > 0.05; Fig. 2B,C).

**Figure 3:**
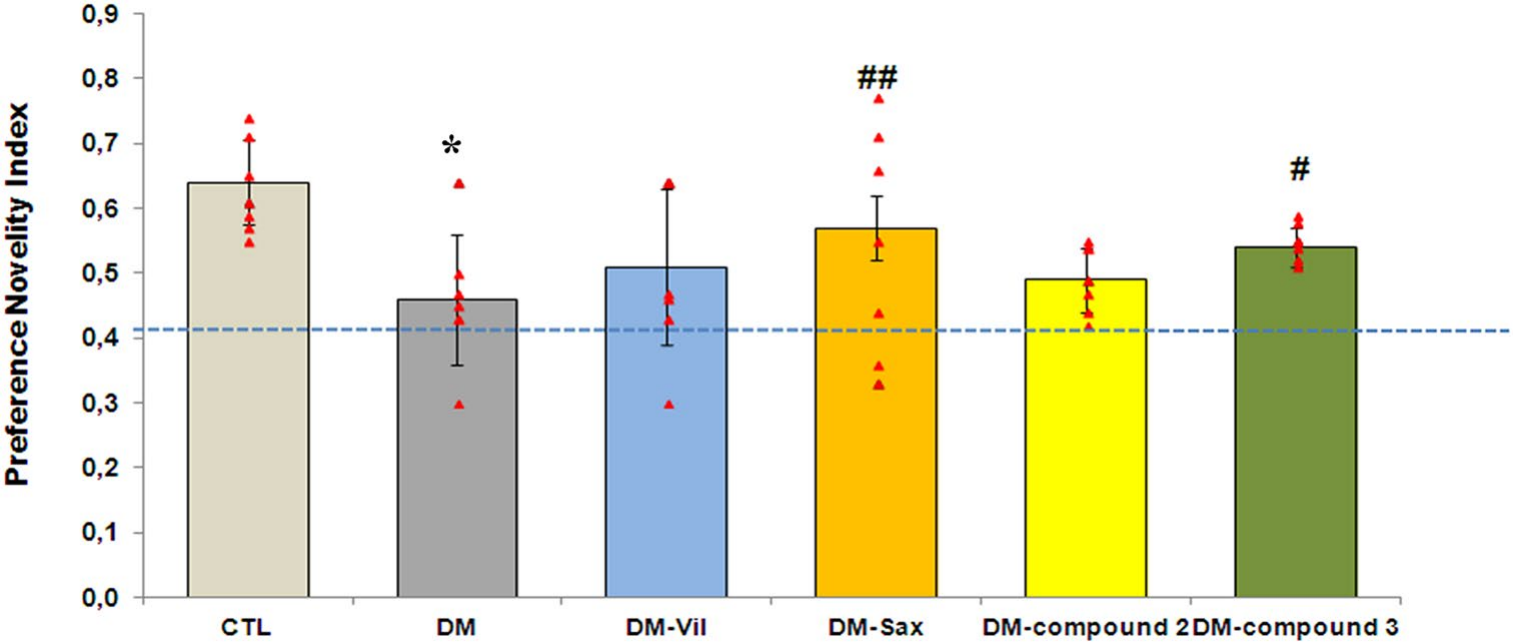
Treatment with novel adamantane derivatives (**2**, **3**) or drugs—vildagliptin and saxagliptin attenuates memory impairment in induced diabetic mice in Y Maze Novelty Preference test (NPT). The dotted line at 0.5 indicates the chance performance value. Scores greater than 0.5 indicate a novelty preference. Bars in figure illustrates experimental groups—*CTL* non-diabetic mice, *DM* diabetic mice, *DM-Vil* vildagliptin-treated mice [20 mg/kg, *po*], *DM-Sax* saxagliptin-treated mice [10 mg/kg, *po*], *DM-compound 2* compound 2-treated mice [50 mg/kg, *po*], *DM-compound 3* compound 3-treated mice [50 mg/kg, *po*]; respectively. Each column represent means ± SD (n = 8). One-way ANOVA, followed by Tukey's post hoc test was used. *p < *0.05* as compared with healthy control group. ^#^*p* < 0.05; ^##^p < 0.01 compared with diabetic mice group.

The effect of novel adamantane derivatives (**2**, **3**) or drugs—vildagliptin and saxagliptin was also checked on the diabetes-induced memory impairments in Y Maze Novelty Preference test (NPT). Saxagliptin- or compound **3**-treated mice showed a relatively higher NP index than untreated animals (95% CI [44.94, 70.56], **p < 0.01, F = 2.931; 95% CI [52.035, 56.465], *p < 0.05, F = 2.931; Fig. 3, respectively) which points to novelty preference by mice. Moreover, the animals treated with the above-mentioned compounds showed a higher preference novelty index than vildagliptin- or compound **2**-treated mice (Fig. 3). The preference novelty index for DM-Vil and DM-compound **2** group was within 0.5. Therefore, the mice did not show a novelty preference, because they explored all arms of a maze at the similar level (*p* > 0.05; Fig. 3). Perhaps this was due to the fact that the tests in the Y-maze were conducted close to each other.

**Figure 4:**
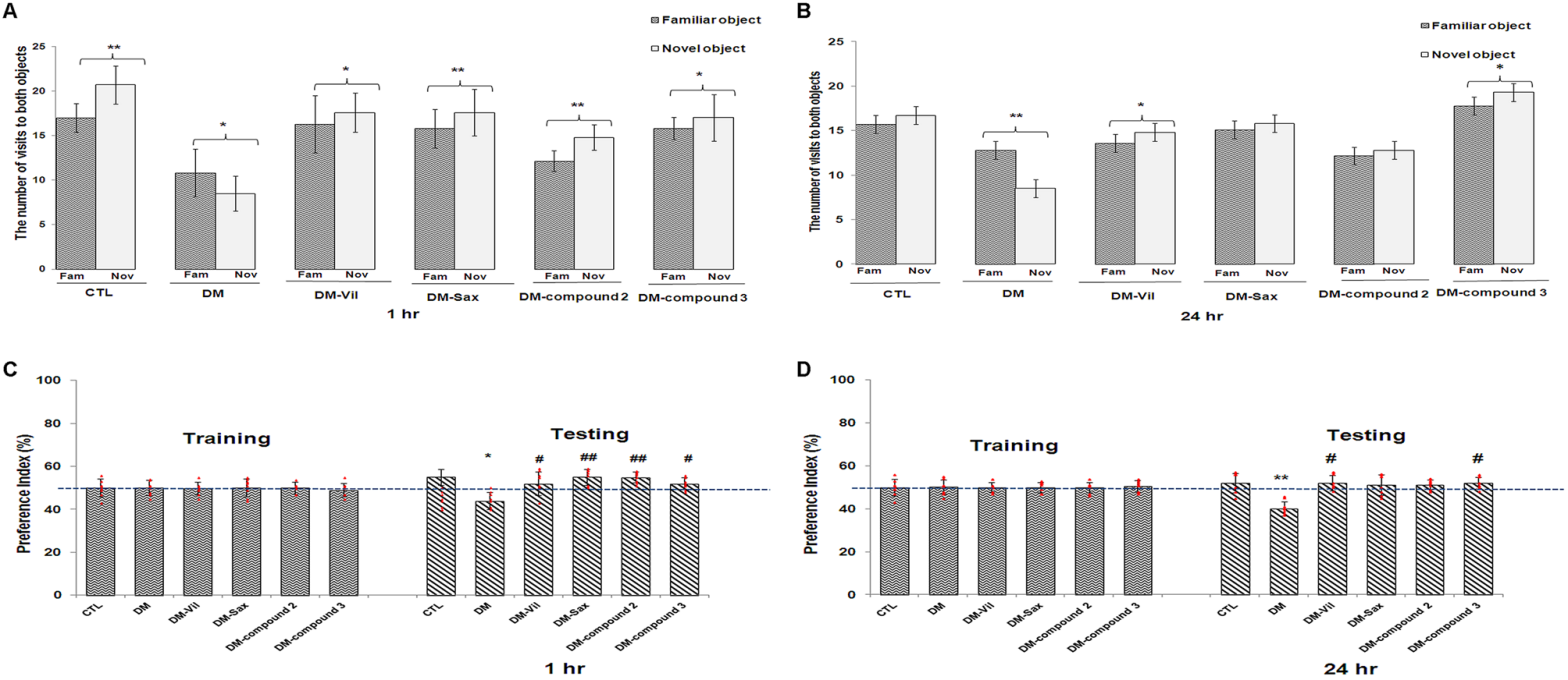
Treatment with novel adamantane derivatives (**2**, **3**) or drugs—vildagliptin and saxagliptin, attenuates memory impairment in induced diabetic mice in novel object recognition (NOR) test (**A**–**D**). Novel adamantane derivatives (**2**, **3**) or vildagliptin and saxagliptin were administered (on the last day 2-weeks experiment) 1 h before the training session. Testing sessions were performed 1 h (**A**,**C**), or 24 h (**B**,**D**) after the training session. The recognition memory was measured by the novel object recognition test where the mice were allowed to explore the familiar (Fam) and novel (Nov) objects for 5 min. Bars in the figures (**A**,**B**) illustrates the number of visits to both objects by different groups of mice in testing stage; bars in figures (**C**,**D**) illustrates the preference index (%) for each group. Experimental groups—*CTL* non-diabetic mice, *DM* diabetic mice, *DM-Vil* vildagliptin-treated mice [20 mg/kg, *po*], *DM-Sax* saxagliptin-treated mice [10 mg/kg, *po*], *DM-compound 2* compound 2-treated mice [50 mg/kg, *po*], *DM-compound 3* compound 3-treated mice [50 mg/kg, *po*], respectively. Each column represent means ± SD (n = 8). (**A**,**B**) *p < 0.05; **p < 0.01 comparison of the exploration count of a new object to the familiar object by the mice in a given experimental group. (**C**,**D**) *p < 0.05; **p < 0.01 compared with healthy control group. (**C**,**D**) ^#^*p* < 0.05; ^##^p < 0.01 compared with diabetic mice group.

In order to confirm the effect of novel adamantane derivatives (**2, 3**) or drugs—vildagliptin and saxagliptin on the diabetes-induced memory impairments, novel object recognition (NOR) test was also performed. The statistical analysis showed that mice with induced diabetes showed a significant reduction in their interest of the objects in testing stage, especially of a new one, compared to the animals in control group (95% CI [7.066, 9.934], *p < 0.05, F = 1.667; 95% CI [7.450, 9.550], **p < 0.01, F = 1.555; Fig. 4A,B, respectively).

After both, 1 h and 24 h, when one of the familiar objects was replaced by a novel object, the preference index (%PI) in diabetic mice was significantly below 50% (95% CI [41.731, 47.019], *p < 0.05, F = 13.707; 95% CI [38.535, 42.965],**p < 0.01, F = 13.294; Fig. 4C,D, respectively). Such a result indicates no preference for the novel object by the animals. It is worth mentioning that the performed statistical analysis did not indicate the preference of the object in the training stage in none of the experimental groups. Animals spent a similar amount of time testing the same objects. The preference index was 50% (Fig. 4C,D). 14 days treatment with vildagliptin, saxagliptin or new derivatives (**2**, **3**) showed an increased exploratory activity of mice directed to the new object after the first hour from the completed training (95% CI [16.101, 19.149], *p < 0.05, F = 2.229; 95% CI [15.773, 19.474], **p < 0.01, F = 1.489; 95% CI [12.906, 15.344], **p < 0.01, F = 1.566; 95% CI [14.810, 18.190], *p < 0.05, F = 2.408; Fig. 4A, respectively). The preference index (%PI) in treated groups were above 50% which indicated a preference of animals to novel object (95% CI [49.088, 56.412], ^#^p < 0.05, F = 13.707; 95% CI [51.903, 58.097], ^##^p < 0.01, F = 13.707; 95% CI [52.711, 57.039], ^##^p < 0.01, F = 13.707; 95% CI [49.577, 54.173], ^#^p < 0.05, F = 13.707; Fig. 4C, respectively). Also, testing session conducted in mice 24 h after the training stage, showed also PI above 50% (Fig. 4D). The greatest preference of animals to novel object was observed in groups treated with vildagliptin or compound 3 (95% CI [49.862, 55.638], ^#^*p* < 0.05, F = 13.294; 95% CI [50.055, 54.695], ^#^p < 0.05, F = 13.294; Fig. 4D, respectively).

Whereas, a slightly lower preference index was observed in mice treated with saxagliptin or compound 2 (*p* > 0.05; Fig. 4D). The preference index (%PI) in the above-mentioned groups was within 50%. The mice did not show the preference of the object, they explored both objects at a similar level (*p* > 0.05; Fig. 4B).

The performance of mice for short- and long-term memory was assessed also with the use of the passive avoidance task. The latency during the learning trial did not differ among any of the groups (data not shown), indicating that all the mice had similar responses to the testing environment and electric shocks.

**Figure 5:**
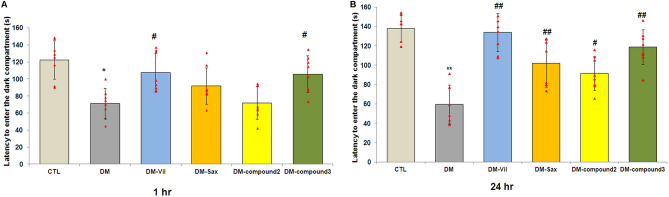
Treatment with novel adamantane derivatives (**2**, **3**) or drugs—vildagliptin and saxagliptin, attenuates memory impairment in induced diabetic mice in passive avoidance test (PA). Retention test was performed after 1 h (**A**) and 24 h (**B**) after the end of the training stage. The bars illustrate the latency to enter the dark compartment of apparatus in testing stage for experimental groups—*CTL* non-diabetic mice, *DM* diabetic mice, *DM-Vil* vildagliptin-treated mice [20 mg/kg, *po*], *DM-Sax* saxagliptin-treated mice [10 mg/kg, *po*], *DM-compound 2* compound 2-treated mice [50 mg/kg, *po*], *DM-compound 3* compound 3-treated mice [50 mg/kg, *po*], respectively. Each column represent means ± SD (n = 8). Repeated-measures ANOVA, followed by Tukey's post hoc test was used. (A-B) *p < 0.05 ; **p < 0.01 compared with healthy control group. (**A**,**B**) ^#^p < 0.05; ^##^p < 0.01 compared with diabetic mice group.

Figure 5A,B shows significant differences in the latency to enter the dark compartment between healthy and diabetic animals in testing session. Animals with induced diabetes showed a significantly shorter latency to enter the dark part of the apparatus both after an hour and 24 h after the training process when compared with non-diabetic mice (95% CI [57.210, 85.763], *p < 0.05, F = 7.833; 95% CI [42.770, 76.290], **p < 0.01, F = 18.966; Fig. 5A,B, respectively). In addition, it is also worth noting that the passage of time from the testing session was dramatically decreased the step-through latency in diabetic mice. The reduced latency in diabetic mice was successfully restored by oral administration of tested compounds. The studied compounds (except compound **2** and saxagliptin; the results statistically non-significant 1 h after the training session, (*p* > 0.05) significantly extended the time needed to enter the dark part of the apparatus compared to the diabetic group (Fig. 5A). Vildagliptin increased the time needed to enter into the dark compartment of treated mice to almost twofold compared to the diabetic group (95% CI [88.363, 126.34], ^#^*p* < 0.05, F = 7.833; Fig. 5A). A similar result was observed in diabetic mice after 14-days treatment with compound **3** (95% CI [87.631, 123.66], ^#^*p* < 0.05, F = 7.833; Fig. 5A). These compounds made possible to achieve a reaction time similar to that which was achieved by healthy mice from the control group. Similar enhancements of retention time-latency was observed in treated mice vildagliptin or compound 3, 24 h after testing session (95% CI [118.10, 150.19], ^##^*p* < 0.01, F = 18.996; 95% CI [102.90, 133.34], ^##^*p* < 0.01 F = 18.996; Fig. 5B, respectively).

Based on the obtained results, it can be concluded that compound **3** and vildagliptin could effectively improve both short- and long-term memory disorders caused by diabetes. It is most noticeable 24 h after the training process. Vildagliptin-administered group exhibited almost similar mean time-latency as the control group. 14-days administration of both saxagliptin and compounds **2** had no statistically significant effect on latency time during the retention test performed after an hour (*p* > 0.05; Fig. 5A).

### Effect of treatment with novel adamantane derivatives and drugs: vildagliptin and saxagliptin on GLP-1 and DPP4 protein levels in prefrontal cortex of mice

**Figure 6:**
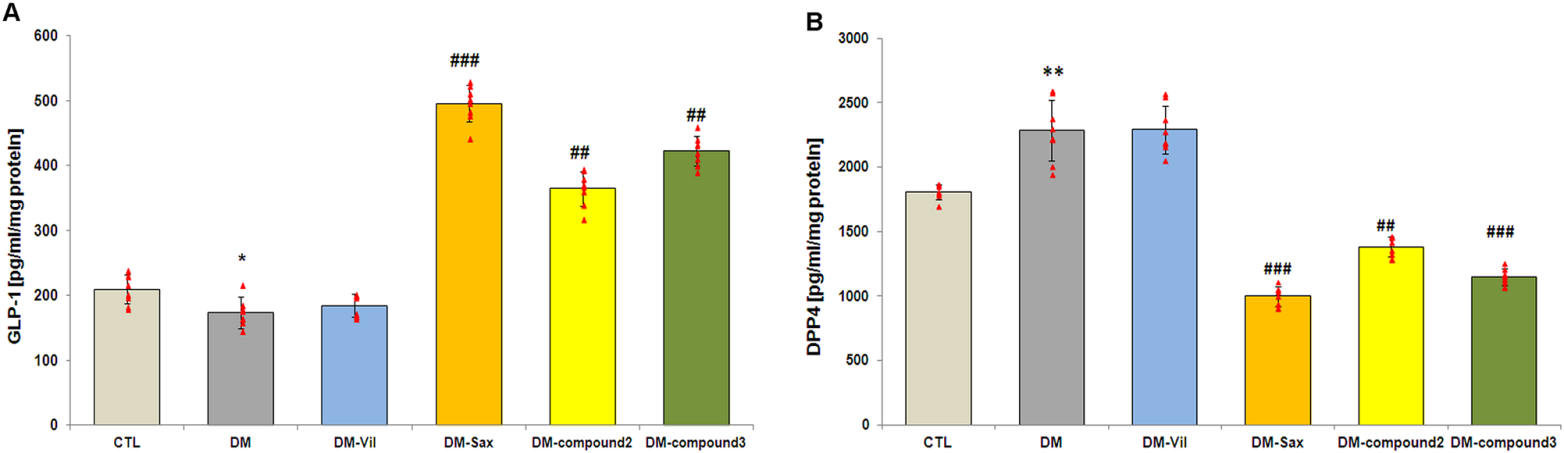
Effect of derivatives (**2**, **3**) and vildagliptin, saxagliptin on the protein level of GLP-1 and DPP4 in prefrontal cortex of diabetic mice. GLP-1 (**A**) and DPP4 (**B**) protein levels in prefrontal cortex tissues of mice after 14-days treatment with DPP41s or new adamantane derivatives (**2**, **3**). Bars in figure illustrates experimental groups—*CTL* non-diabetic mice, *DM* diabetic mice, *DM-Vil* vildagliptin-treated mice [20 mg/kg, *po*], *DM-Sax* saxagliptin-treated mice [10 mg/kg, *po*], *DM-compound 2* compound 2-treated mice [50 mg/kg, *po*], *DM-compound 3* compound 3-treated mice [50 mg/kg, *po*], respectively. Data is presented as mean ± SD (n = 8). One-way ANOVA, followed by Tukey's post hoc test was used. *p < 0.05; **p < 0.01 as compared with healthy control group; ^##^p < 0.01, ^###^p < 0.001 compared with diabetic mice group.

The GLP-1 protein level in prefrontal cortex of diabetic mice was significantly lower compared to the control group (non-diabetic mice; 95% CI [156.6, 191.64],*p < 0.05, F = 277.01; Fig. 6A). Whereas, DPP4 protein levels were significantly higher in prefrontal cortex of diabetic mice compared to the control group (95% CI [2080.7, 2471.1], **p < 0.01, F = 139.51; Fig. 6B).

Mice treated with saxagliptin or new adamantane derivatives **2** and **3** for 14 weeks exhibited a significant increase in GLP-1 protein level in prefrontal cortex compared with diabetic mice (95% CI [471.34, 518.89], ^###^*p* < 0.001, F = 277.01; 95% CI [342.87, 386.83], ^##^*p* < 0.01, F = 277.01; 95% CI [403.76, 441.79], ^##^*p* < 0.01, F = 277.01, Fig. 6A, respectively). The level of GLP-1 protein was further associated with the level of DPP4 release in the examined tissues (Fig. 6A,B). Mice treated with saxagliptin or new adamantane derivatives for 14 days exhibited reductions in DPP4 protein release in the examined tissues (95% CI [937.42, 1063.0], ^###^*p* < 0.001, F = 139.51; 95% CI [1316.8, 1448.4], ^##^*p* < 0.01; F = 139.51; 95% CI [1095.9, 1202.3], ^###^*p* < 0.001, F = 139.51, appropriately; Fig. 6B) what leads to significant increase of brain GLP-1 level (Fig. 6A). It was recorded as much as threefold increase of GLP-1 in mice treated with saxagliptin (95% CI [471.34, 518.89], ^###^*p* < 0.001, F = 277.01; Fig. 6A), twofold increase after applying compound **2** (F = 277.01; 95% CI [342.87, 386.83], ^##^*p* < 0.01, F = 277.01; Fig. 6A) and 2.5-fold increase of this parameter after 14-days treatment with derivative **3** (95% CI [403.76, 441.79], ^##^*p* < 0.01, F = 277.01;Fig. 6A). Whereas, two weeks-treatment of diabetic mice with vildagliptin had no effect on these parameters (*p* > 0.05; Fig. 6A,B).

### The novel adamantane derivatives (2, 3) and DPP4 inhibitors limited the diabetes-induced production of pro-inflammatory cytokines in prefrontal cortex of mice

**Figure 7:**
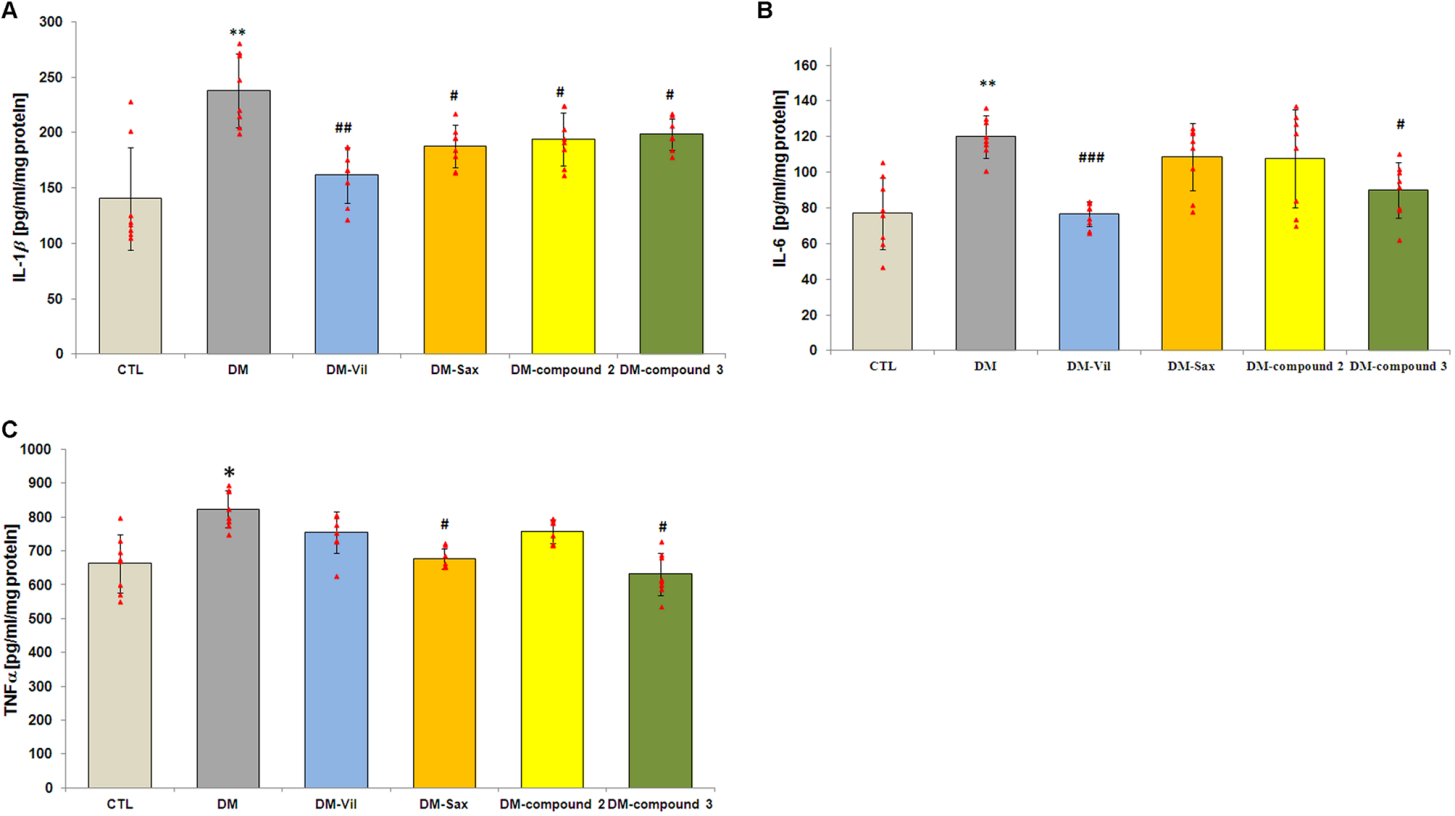
Anti-inflammatory effects of novel adamantane derivatives (2,3), and drugs- vildagliptin and saxagliptin in brain of diabetic mice. Comparison of IL-1β (**A**), IL-6 (**B**), and tumor necrosis factor-α (TNFα) (**C**) among the experimental groups. Bars in figure illustrate experimental groups—*CTL* non-diabetic mice, *DM* diabetic mice, *DM-Vil* vildagliptin­treated mice [20 mg/kg, *po*], *DM-Sax* saxagliptin-treated mice [10 mg/kg, *po*], *DM-compound 2* compound 2-treated mice [50 mg/kg, *po*], *DM-compound 3* compound 3-treated mice [50 mg/kg, *po*], respectively. Data is presented as mean ± SD (n = 8). One-way ANOVA, followed by Tukey's post hoc test was used. *p* < 0.05, **p < 0.01 in comparison with control group; ^#^*p* < 0.05 and ^##^p < 0.01, ^###^p < 0.001 in comparison with diabetes group.

The results showed that the protein levels of cytokines: IL-1*β*, IL-6 and, TNF-*α* in prefrontal cortex of the diabetes mice were significantly higher when compared to the control group (95% CI [211.22, 266.28], **p < 0.01, F = 10.962; 95% CI [111.04, 129.46], **p < 0.01, F = 8.268; 95% CI [778.84, 868.66], *p < 0.05, F = 12.793; Fig. 7A–C, respectively). The levels of interleukin 1β and interleukin 6 were from 1.5 to 1.7 times higher in the prefrontal cortex of diabetic mice than in control mice (Fig. 7A,B). In turn, the level of tumor necrosis factor-α was clearly higher (about 24%) in the prefrontal cortex of diabetic mice than in the control group (Fig. 7C). In all treated mice, the reductions of IL-1*β*, IL-6 and TNF-α protein level were observed (Fig. 7A–C). However, the levels of Interleukin 1*β* and IL-6 were the most decreased after 14-days treatment with vildagliptin. An almost 35% decrease of the level of these cytokines was recorded in the prefrontal cortex of diabetic mice compared to untreated animals (95% CI [141.58, 181.92], ^##^*p* < 0.01, F = 10.962; 95% CI [69.876, 81.624], ^###^*p* < 0.001, F = 8.268; Fig. 7A,B, respectively).

In turn, the anti-inflammatory activity of the newly synthesized adamantane derivative (compound **2**) and saxagliptin seemed to be at a comparable level. Both compounds led to a moderate reduction in the level of interleukin 1β by 19% and 17%, respectively (95% CI [174.80, 213.45], ^#^*p* < 0.05, F = 10.962; 95% CI [172.47, 203.28], ^#^*p* < 0.05, F = 10.962; Fig. 7A) and had no statistically significant effect on the level of IL-6 (*p* > 0.05; Fig. 7B).

It should be highlighted, that compound **3** significantly decreased the level of all tested cytokines in prefrontal cortex of the diabetes mice (Fig. 7A–C). The levels of IL-6 and TNF-α cytokines were lowered by almost 25% (95% CI [77.022, 103.23], ^#^*p* < 0.05, F = 8.268; 95% CI [579.30, 684.70], ^#^*p* < 0.05, F = 12.793; Fig. 7B,C, respectively) and IL-1β (95% CI [186.96, 210.54], ^#^*p* < 0.05, F = 10.962; Fig. 7A) by 16% compared to the level of these parameters in diabetic mice.

### The effect of treatment of novel adamantane derivatives (2,3), and drugs: vildagliptin and saxagliptin on *Bdnf* and *Cav1* genes expression in the hippocampus of diabetes mice

**Figure 8:**
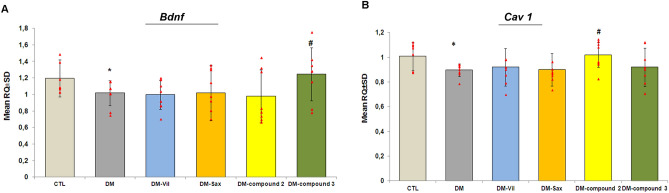
The effects of novel adamantane derivatives (2, 3) and DPP4 inhibitors on *Bdnf* (A) and *Cav1* (B) genes expression in the hippocampus isolated from diabetes mice. Bars in figure illustrate experimental groups—*CTL* non-diabetic mice, *DM* diabetic mice, *DM-Vil* vildagliptin-treated mice [20 mg/kg, *po*], *DM-Sax* saxagliptin-treated mice [10 mg/kg, *po*], *DM-compound 2* compound 2-treated mice [50 mg/kg, *po*], *DM­compound 3* compound 3-treated mice [50 mg/kg, *po*], respectively. Data is presented as mean ± SD (n = 8). One-way ANOVA, followed by Tukey's post hoc test was used. *p < 0.05 in comparison with control group; ^#^p < 0.05 in comparison with diabetes group.

In order to further elucidate the molecular mechanisms underlying the memory enhancing effect of novel adamantane derivatives (**2**, **3**) and DPP4 inhibitors, we have examined the gene expression levels of *Bdnf* and *Cav1* in the hippocampal tissues of diabetes mice. The real time RT-PCR study showed the statistically significant decrease in *Bdnf* and *Cav1* expression levels in the brain of diabetes mice compared to control group (95% CI [0.8708, 1.133], *p < 0.05, F = 1.484; 95% CI [0.8545, 0.9418], *p < 0.05, F = 1.538; Fig. 8A,B, respectively). Our data showed that 14-days treatment of diabetes mice with compound **3** (50 mg/kg, *po*) significantly increased *Bdnf* gene expression in the hippocampus of mice compared to untreated group (95% CI [0.9662, 1.496], *p < 0.05, F = 1.484; Fig. 8A), whereas other compounds did not significantly affect the expression level of the studied gene (*p* > 0.05). In turn, a moderate higher expression of *Cav1* mRNA level was observed in the hippocampus of animals treated for 14 days with compound **2** when compared to untreated mice (95% CI [0.9260, 1.114], *p < 0.05, F = 1.538; Fig. 8B). This might imply neuroprotective effects of new adamantane derivatives in diabetes mice. The remaining tested compounds had no significant effect on the expression of the *Cav1* gene in diabetes mice (*p* > 0.05).

### The correlation between interleukin levels in the prefrontal cortex of diabetic mice and the results of behavioral tests

There was a significant positive, very high linear correlation between the level of interleukin 6 (r = 0.788, *p < 0.05), TNF*α* (r = 0.817, *p < 0.05) in prefrontal cortex and cognitive impairment in diabetics mice in Y Maze Novelty Preference Test (NPT). Also, a significant positive high linear correlation between the level of TNF*α* (r = 0.753, *p < 0.05), interleukin 1*β* (r = 0.739, *p < 0.05) in prefrontal cortex and long-term memory impairment in diabetic mice in passive avoidance test (PA) was observed.

## Discussion

Diabetes is a chronic disease which may lead to cognitive impairment like memory difficulties, deterioration of learning abilities and psychomotor slowing. Many factors may be involved in the pathogenesis of the above-mentioned disorders, such as the hypoglycemic and hyperglycemic episodes, cerebrovascular alterations, insulin-signaling disorders and oxidative stress^60–65^. Under oxidative stress, excessive production of reactive oxygen species (ROS) activate NF-κB, activator protein-1 (AP-1), and STAT pathways which in turn, leads to the release of inflammatory cytokines^3,6,66,67^. Studies showed that chronic inflammation in hippocampus and frontal cortex of the brain can promote not only the release of pro-inflammatory cytokines, but also highly toxic products such as reactive oxygen intermediates, nitric oxide (NO) or proteolytic enzymes which in effect act destructively on neurons^68,69^. Brain imaging studies of diabetic patients revealed changes in various brain regions such as hippocampus and frontal cortex, as well as, a marked reduction in grey matter density and a reduction in white matter which was related with cognitive decline like poorer visuospatial construction, memory, or locomotor activity^70–74^.

Recently, in the published article we confirmed a close relationship between hyperglycemia, hyperinsulinemia and high expression of inflammatory cytokines in brain, and cognitive dysfunction in T2DM mouse model^9^. The overproduction of pro-inflammatory cytokines in prefrontal cortex in diabetes mice was associated with lower locomotor and cognitive activity of animals and it can indicate neuronal disturbances in this area of the brain. Additionally, the results presented in our article proved that an impairment in terms of attention, recognition and spatial memory in the studied time intervals may point to progression of cognitive disorders in CNS in model animals. Similarly, Ramos-Rodriguez et al*.*^75^ and Flood et al*.*^76^ confirmed deficits in spatial memory and learning in diabetic mice. Roghani et al*.*^77^ showed that STZ-induced diabetic rats show shorter latency in the passive avoidance test and deficits in working memory in the Y-maze test. Jabbarpour et al*.*^78^ draw similar conclusions and they confirmed deterioration in spatial memory performance and learning ability in diabetes mice. Similarly, Wang et al*.*^3^ showed that old diabetic rats exhibited impaired spatial learning and short- and long-term memory without damaged locomotor activity and exploratory behaviours. These deteriorations were plausibly associated with the significantly increased BBB leakage in the cortex and hippocampus, as well as impaired cerebral blood flow, decreased number of neurons, and overall neurodegeneration. In turn, in young diabetic rats BBB leakage was not detected, but these rats also showed neurodegeneration^3^.

Beside this, diabetes-induced memory impairment was also accompanied by the decrease of *Bdnf* and *Cav1* gene expression levels in the hippocampal tissues, which could have had a significant impact on the extent and intensity of the inflammation and brain lesion volume as reported also by the other authors^47,79,80^. Wu et al*.*^48^ have showed that cognitive deficits in diabetes rats are directly related with decrease of *Cav1* expression, increase of tau phosphorylation and activate mTOR/S6K signaling in brain. The analysis of *Cav1* expression performed in frozen brain sections of T2DM patients by the Bonds et al*.* confirmed lower gene expression involved in the inflammatory response of brain and higher amyloid-β levels versus healthy people^50^. The aforementioned studies seem to support the idea that Alzheimer’s disease may be a complication of T2DM what has been recently proposed. This hypothesis is also sustained by the evidence that insulin resistance leads to stress at mitochondrial level causing the apoptosis of neurons and neuroinflammation^81^.

The research conducted by our group confirmed not only the correlation between the level of pro-inflammatory interleukins and cognitive impairment of diabetes mice but also a significant involvement in CNS functions of GLP-1-glucagon-like peptide (GLP-1). The GLP-1 as a growth factor in the brain can not only induce neurons growth but it also can regulate glucose metabolism, limit insulin resistance, as well as decrease the level of inflammatory molecules through various mechanisms, dependent or independent on receptor (GLP1R)^82–85^. Our research showed that mice with diabetes mellitus had lower GLP-1 levels than healthy animals and thus higher level of pro-inflammatory cytokines in prefrontal cortex. Such a link can lead to the progression of inflammation and it can affect the severity of the disorder in central nervous system^86^. Experimental studies of Isacson et al*.*^87^ confirmed that GLP-1 receptor knockout mice exhibit impaired learning and memory and disturbed synaptic transmission in the hippocampus.

A lot of research showed that GLP-1 mimetic drugs or GLP-1 availability modulating drugs as DDP4 inhibitors have neuroprotective, neurotrophic, and anti-inflammatory effects, which can play important role in retardation of cognitive impairment progression^38–41,43,44,86,88–90^. Many studies also proved that increased level of the GLP-1 reduces the expression of the pro-inflammatory cytokines (TNF-*α*, IL-1*β* and IL-6) thereby it limits the extent of inflammation^3,65,68,69^. The results of our research also confirmed this. We demonstrated that GLP-1 is an essential molecule for the suppression of diabetes-induced inflammation by DPP4 inhibitors.

Novel compounds tested by our research group raised the level of GLP-1 protein which resulted in the reduction of pro-inflammatory cytokines in prefrontal cortex of mice and hence an improvement of cognitive activity of mice was observed. We demonstrated that central blockade of pro-inflammatory cytokines synthesis significantly improved memory performance in diabetes mice. Newly-synthesized compounds improved the recognition memory outcomes measured by Y Maze Novelty Preference test (NPT) and NOR tasks and spatial memory in Y Maze test. Moreover, in the passive avoidance task, we noticed that there was an improvement in memory consolidation in mice treated specifically with vildagliptin or derivative **3**. The mechanism by which these compounds facilitate consolidation of memory may arise from stimulation of protein synthesis related to plasticity of brain^91^. An extensive literature review implicates that synaptic plasticity in the acquisition, consolidation and long-term storage of different types of memory may depend on gene expression and protein synthesis such as brain-derived neurotrophic factor (*Bdnf*) or caveolin-1 (*Cav1*)^92,93^. Our work showed that among the tested compounds, derivative **3** turned out to be the most effective, while derivative **2** or vildagliptin and saxagliptin were slightly less effective. After 14 days of treatment of diabetes mice with derivative **3** (50 mg/kg *po*) the mRNA expression level of *Bdnf* gene was significantly increased in the hippocampus, which was associated with cognitive improvement in mice.

The in vitro study by Wang et al*.*^94^ showed that the overexpression of *Cav1* in murine macrophages dramatically inhibited TNF-*α* and IL-6 production and increased the secretion of anti-inflammatory interleukin 10. Moreover, it has been proved that *Cav1* overexpression decreases HGC-induced tau hyperphosphorylation in the hippocampal primary neurons^48^. Bonds et al*.*^50^ confirmed that restoration of *Cav1* expression in diabetic mice restored the learning deficiency.

The results of our research were consistent with the observations of other scientists, we indicated that diabetes mice receiving daily for 14 days novel adamantane derivative **2** exhibited significantly higher expression level of hippocampal *Cav1* and the reduction of memory and learning impairments. These findings offer important proof-of-concept evidence for the use of compounds modulating *Cav1* expression at early stages of cognitive loss.

Moreover, our research has shown that anti-inflammatory and neuroprotective mechanism of the novel adamantane derivatives may be also related to the modulation of DPP4 and thus the availability of GLP-1 protein in the brain of treated animals. More and more research showed that increase of the GLP-1 availability in CNS can promote neuronal survival and ameliorate cognitive deficits^6,66,67^. In mice treated with a GLP-1 receptor agonists an increase in the number of neurons in the hippocampus was observed and, consequently, an improvement in cognitive skills^16^. Although, the exact mechanism of these positive actions of GLP-1 protein on cognition has yet to be understood it presumably may involve GABA-A receptor-signaling^38–41,69,82,87,95^ and/or may be associated with an improvement of insulin signaling pathway^95^. So a unique mechanism of action of DPP4 inhibitors may be used to improve the cognitive skills in the AD and other neurological diseases.

El-Sahar et al*.*^43^ and Újhelyi et al*.*^44^ confirmed the anti-inflammatory effects of two DPP4 inhibitors: sitagliptin and vildagliptin in mouse model of inflammation. In the above-cited studies, drugs effectively reduced the level of pro-inflammatory markers in the hippocampus and in the inflamed tissues. Satoh-Asahara et al*.*^45^ also confirmed an anti-inflammatory effects of sitagliptin. The therapy with this drug not only lowered the level of pro-inflammatory markers such as C-reactive protein (CRP) or TNF-α, but also increased levels of anti-inflammatory cytokines which ultimately led to reduce the inflammation in the blood serum of patients with diabetes. In turn, Makdissi et al*.*^34^ showed that sitagliptin, apart from a significant reduction of TNF-α and IL-6 level, simultaneously increased the fasting blood level of GLP-1 in obese diabetic patients.

In turn, in a study conducted on rats with streptozotocin-induced diabetes mellitus, vildagliptin was proved to have a beneficial effect not only on reducing blood glucose level but also on improving learning and memory in animals, as demonstrated by the NOR test. Animals treated with vildagliptin showed greater interest in the new object than in the previously known one^89^.

Summarizing, the results of the conducted studies confirmed the neuroprotective effects of newly synthesized adamantane derivatives and dipeptidyl peptidase 4 inhibitors in diabetes mice. Among the tested compounds, derivative **3** turned out to be the most effective, while the derivative **2** and saxagliptin and vildagliptin were slightly less effective. The varied efficacy of the tested compounds seems to depend on the degree of inhibition of DPP4 protein release and the GLP-1 protein level in the CNS. We found that the neuroprotective effects of newly synthesized adamantane derivatives and saxagliptin may be attributed to modulation of the GLP-1 signaling pathways in CNS. We demonstrated that GLP-1 overexpression in the prefrontal cortex translates into a central blockade of pro-inflammatory cytokines synthesis and significantly improves memory performance especially in diabetes mice treated with newly synthesized adamantane derivative **3** or saxagliptin. Therefore, these findings suggest that high level of GLP-1 may be a key indicator of regulation of neuroinflammation and it may also act to improve cognitive abilities and memory in adamantane derivatives-treated animals. Therefore, it can be concluded that brain GLP-1 may play a key role in development of neuropathological changes as well as it could be an attractive therapeutic target. Newly synthesized adamantane derivatives or DPP4 inhibitors might have important roles in the future in prevention and treatment of cognitive impairment caused by inflammatory events in patients with diabetes or related diseases.

It may be that, in the future the proposed compounds will be able to play an important role also in limitation of CNS complications in patients recovered from COVID-19. The cognitive impairment (problems with memory, attention, and thought processing) known as "brain fog" is now widely reported in COVID-19 survivors after several months from diagnosis. Nevertheless, further research in this area is needed. The extensive studies on adamantane derivatives in this area would undoubtedly be a valuable contribution (Supplementary Information S1).

## Supplementary Information

Supplementary Information.
