# Supplementary material for: The novel adamantane derivatives as potential mediators of inflammation and neural plasticity in diabetes mice with cognitive impairment
Source: Sci Rep. 2022 Apr 25;12:6708. doi: 10.1038/s41598-022-10187-y (PMC9035983; doi:10.1038/s41598-022-10187-y)
Supplement: Supplementary file 1 — Supplementary Information. [file 41598_2022_10187_MOESM1_ESM.docx]

***Supplementary information***

**S1.** In this method a competitive reaction is used between biotin-labelled and unlabeled GLP-1 to limited amount of monoclonal antibodies specific to GLP-1 which has been pre-coated onto a microplate by the manufacturer of kit. After 1 hour of incubation at 37°C with reagent A, unbound antibodies were washed away and a reagent B, which is a secondary antibody-conjugate with horseradish peroxidase (HRP), was added. After 30 minutes of incubation with reagent B and washing several times, a substrate solution - tetramethylbenzidine (TMB) was added. During this time, a chemical reaction catalyzed by the HRP enzyme, takes place. After 20 minutes, the reaction was terminated by adding the stop solution. The amount of reaction product formed was measured spectrophotometrically at a wavelength of 450 nm immediately using a microplate reader (Bio Tek, Elx808, Poland). The signal intensity was reverse proportional to the GLP-1 content in the determined sample. The results were compared to the standard curve. GLP-1 concentrations in brain samples were then normalized to the total protein concentration determined for each sample. The results are expressed in pg /mg protein.

**S2.** First, detection reagent A was added to the wells of microplate which was next incubated 1 hour at 37°C. Then, the wells of microplate was washed with buffer. The detection reagent B was added and the plate was incubated next 30 minutes at 37°C. After this time, TMB substrate solution was added and it was stored away from light for 20 minutes. A colour change to blue was observed as a result of a reaction catalysed by the horseradish peroxidase (HRP) enzyme. After the expected time has elapsed, the stop solution (sulfuric acid solution) was added and a colour change to yellow was observed. The colour intensity was measured at a wavelength of 450 nm using a spectrophotometric microplate reader (BioTek, Elx808, Poland). The concentration of cytokines in samples were determined by comparing the optical density of the samples to the individual standard curve.

The signal intensity was proportional to the pro-inflammatory cytokine quantity in the determined sample. Cytokine concentrations in brain samples were expressed in picograms per mg of protein.
